# High expression of LOC541471, GDAP1, SOD1, and STK25 is associated with poor overall survival of patients with acute myeloid leukemia

**DOI:** 10.1002/cam4.5644

**Published:** 2023-01-27

**Authors:** Xibao Yu, Cunte Chen, Yanyun Hu, Kehan Li, Yikai Zhang, Zheng Chen, Dingrui Nie, Rili Gao, Youxue Huang, Mengjun Zhong, Caixia Wang, Shunqing Wang, Yixin Zeng, Yangqiu Li, Chengwu Zeng

**Affiliations:** ^1^ The First Affiliated Hospital, Institute of Hematology, School of Medicine, Jinan University Guangzhou China; ^2^ Department of Experimental Research Sun Yat‐sen University Cancer Center, State Key Laboratory Oncology in South China Guangzhou China; ^3^ Guangzhou Municipality Tianhe Nuoya Bio‐engineering Co. Ltd Guangzhou China; ^4^ Department of Hematology Guangzhou First People's Hospital, School of Medicine, South China University of Technology Guangzhou China

**Keywords:** acute myeloid leukemia, apoptosis, biomarker, long non‐coding RNA, nomogram, risk stratification

## Abstract

**Background:**

Acute myeloid leukemia (AML) is an aggressive heterogeneous hematological malignancy with remarkably heterogeneous outcomes. This study aimed to identify potential biomarkers for AML risk stratification via analysis of gene expression profiles.

**Methods:**

RNA sequencing data from 167 adult AML patients in the Cancer Genome Atlas (TCGA) database were obtained for overall survival (OS) analysis, and 52 bone marrow (BM) samples from our clinical center were used for validation. Additionally, siRNA was used to investigate the role of prognostic genes in the apoptosis and proliferation of AML cells.

**Results:**

Co‐expression of 103 long non‐coding RNAs (lncRNAs) and mRNAs in the red module that were positively correlated with European Leukemia Network (ELN) risk stratification and age was identified by weighted gene co‐expression network analysis (WGCNA). After screening by uni‐ and multivariate Cox regression, Kaplan–Meier survival, and protein–protein interaction analysis, four genes including the lncRNA LOC541471, GDAP1, SOD1, and STK25 were incorporated into calculating a risk score from coefficients of the multivariate Cox regression model. Notably, GDAP1 expression was the greatest contributor to OS among the four genes. Interestingly, the risk score, ELN risk stratification, and age were independent prognostic factors for AML patients, and a nomogram model constructed with these factors could illustrate and personalize the 1‐, 3‐, and 5‐year OS rates of AML patients. The calibration and time‐dependent receiver operating characteristic curves (ROCs) suggested that the nomogram had a good predictive performance. Furthermore, new risk stratification was developed for AML patients based on the nomogram model. Importantly, knockdown of LOC541471, GDPA1, SOD1, or STK25 promoted apoptosis and inhibited the proliferation of THP‐1 cells compared to controls.

**Conclusions:**

High expression of LOC541471, GDAP1, SOD1, and STK25 may be biomarkers for risk stratification of AML patients, which may provide novel insight into evaluating prognosis, monitoring progression, and designing combinational targeted therapies.

## Graphical abstract

In this study, we demonstrate that high expression of LOC541471, GDAP1, SOD1, and STK25 may be biomarkers for risk stratification of AML patients, which may provide novel insight into evaluating prognosis, monitoring progression, and designing combinational targeted therapies.

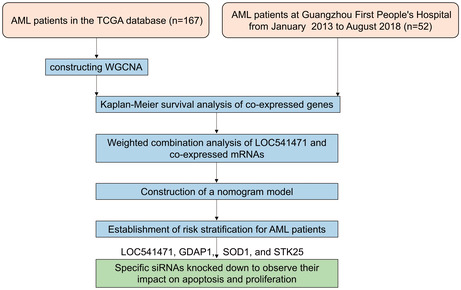

## 1 INTRODUCTION

Acute myeloid leukemia (AML) is a heterogeneous malignant disease with large variations in prognosis, and it is associated with cytogenetic, molecular, and epigenetic aberrations.^1^, ^2^, ^3^, ^4^ AML patients can be divided into low‐, intermediate‐, and high‐risk groups based on their cytogenetics and gene mutations at the time of diagnosis, which is of great significance for monitoring AML patients and selecting appropriate treatment strategies to improve clinical outcomes.^5^, ^6^, ^7^ However, according to the existing risk stratification for treatment options, most patients relapse and die after remission.^8^ The 5‐year overall survival (OS) rate for AML patients is less than 50%, particularly for patients greater than 60 years old whose 5‐year OS rate is less than 20%.^9^ Notably, 40%–50% of patients are classified as intermediate risk.^10^ It is necessary to explore novel biomarkers for risk stratification to perform precise and personalized monitoring better.

Epigenetic alterations have recently been shown to have a key role in the pathogenesis of AML.^11^, ^12^ Long non‐coding RNAs (lncRNAs), as participants in epigenetic regulation, also occupy an indispensable role in the development of cancer.^1^, ^13^ Moreover, in recent years, lncRNAs are shown to be abnormally expressed in a variety of cancer types and are linked to a variety of disease processes and medication resistance.^14^ LncRNA represents a group of non‐coding RNAs with lengths greater than 200 nucleotides that possess limited or no ability to encode proteins, accounting for greater than 80% of the entire transcriptome. Many lncRNAs have been discovered as prognostic biomarkers for AML, including HOTAIRM1, CCDC26, and LOC646762.^1^, ^14^, ^15^

Therefore, in this study, RNA sequencing and clinical data from AML in the Cancer Genome Atlas (TCGA) database were used for analysis of prognostic lncRNAs and mRNAs by weighted gene co‐expression network analysis (WGCNA). The results were further validated in AML bone marrow (BM) samples from our clinical center by quantitative real‐time PCR (qRT‐PCR). Moreover, the impact of lncRNA and co‐expressed mRNA on the apoptosis and proliferation of AML cells was further investigated.

## 2 MATERIALS AND METHODS

### 2.1 TCGA dataset

The RNA sequencing data and complete clinical information of 167 AML patients, which were used for univariate COX regression analysis, were downloaded from TCGA (https://cancergenome.nih.gov/) database^16^, ^17^ using the “RTCGAToolbox” package.^14^, ^18^ We selected genes with *p* < 0.05 and excluded repeat genes. We finally identified 3,684 genes associated with prognosis, and these were included in the construction of WGCNA.^14^, ^19^ TCGA dataset was assigned as a training cohort, and the clinical characteristics are listed in Table S1.

### 2.2 BM samples

Because the samples of 167 AML patients in the TCGA database were derived from BM; BM samples from our clinical center were collected for validation. BM samples from 52 patients diagnosed with de novo AML between January 1, 2013, and August 31, 2018, were obtained from Guangzhou First People's Hospital, and these samples served as a validation cohort. The inclusion criteria were as follows: (I) De novo AML patients diagnosed according to the French–American–British (FAB) classification system and (II) Age ≥ 18 years. Notably, AML patients who died early before chemotherapy were excluded. In addition, BM samples from 12 healthy individuals were used as a control. The date of the last follow‐up was January 6, 2021, and the median follow‐up time for the surviving patients was 1,155 days (range: 5–2422 days). The clinical characteristics of the 52 AML patients are listed in Table S1. OS is defined as the span from the diagnosis of AML to death from any cause. Relapse‐free survival (RFS) is defined as the span from complete remission of AML patients after receiving chemotherapy to relapse or death. This study was approved by the Ethics Committee of the First People's Hospital of Guangzhou, and all participants provided written informed consent.

### 2.3 Construction of a nomogram model

Clinically significant variables, selecting by univariate and multivariate COX regression analysis, were incorporated into the construction of a nomogram model. The “rms” package in R (version 3.5.1) was used for nomogram model analysis.^20^, ^21^ Each patient was given a total point using standard points obtained from the nomogram model, which could predict 1‐, 3‐, and 5‐year OS rates of AML patients. The predictive ability for nomogram model was evaluated by internal and external validation, which was showed by calibration curves and time‐dependent receiver operating characteristic curve (ROC).

### 2.4 Cell culture and reagents

The AML cell line THP‐1 was cultured in RPMI 1640 (Invitrogen) containing 10% fetal bovine serum (Gibco). The cells were cultured in a humidified atmosphere containing 5% CO_2_ at 37°C. Cytarabine (AraC) was purchased from Selleck Chemicals and used at a final concentration of 1 μM.

### 2.5 RNA extraction and quantitative real‐time RT‐PCR

Total RNA isolation and reverse transcription PCR were performed according to the instructions of the manufacturer.^14^, ^22^ Gene expression levels were quantified by quantitative real‐time RT‐PCR (qRT‐PCR) with SYBR Green (TIANGEN),^23^, ^24^, ^25^, ^26^ and 18 S rRNA served as an internal control. The primer sequences used are listed in Table S2. The gene expression levels are presented as 2^−ΔΔCT^.

### 2.6 RNA interference

THP‐1 cells were transfected using the Neon® Transfection System (Invitrogen) with 100 pmol of oligonucleotides in 10 μl reactions. Transfection was performed as described previously.^27^, ^28^ The siRNA sequences used to target LOC541471, GDAP1, SOD1, and STK25 are listed in Table S2. Control siRNA (si‐NC) was purchased from RiboBio.

### 2.7 Apoptosis analysis

Apoptosis was detected by staining with the Annexin‐V‐APC/PI Apoptosis Detection Kit (MultiSciences). Analysis was performed by flow cytometry using the manufacturer's protocol.^29^, ^30^

### 2.8 Cell viability analysis

To assess cell viability, the CCK‐8 kit (Dojindo) was used according to the manufacturer's protocol.^22^ Briefly, THP‐1 transfected cells were plated at a density of 3,000 cells/well in 96‐well plates and cultured in RPMI 1640 medium containing 10% FBS and 1 μM AraC. CCK‐8 reagent was added to the wells at the end of the experiment. After incubation at 37°C for 4 h, the absorbance of each well was determined using a microplate reader at 450 nm. Medium without cells was used as blank.

### 2.9 Statistical analysis

All statistical analysis was performed using SPSS (version 22.0), R (version 4.0.2, https://www.r‐project.org/), and GraphPad Prism software (version 9.0, Inc.) as appropriate. WGCNA was constructed by the “WGCNA” package, and the detailed description can be found in previous publications.^14^, ^31^ Mann–Whitney‐Wilcoxon was used to compare differences between two groups of quantitative variables. Comparison of categorical variables was performed by the chi‐square and Fisher tests as appropriate.

Univariate Cox proportional hazard regression analysis was performed with R package “survival”. Multivariate Cox proportional hazard regression analysis was performed in a backward stepwise manner. Comparisons between groups of Kaplan–Meier curves were performed by the log‐rank test. Correlation coefficients between two genes were obtained by Spearman's method. The area under the curve (AUC) in the time‐dependent ROC curve was determined by the “survivalROC” package. A two‐tailed *p* value < 0.05 was considered statistically significant for all analyses.

## 3 RESULTS

### 3.1 Identification of 103 highly co‐expressed genes

**FIGURE 1:**
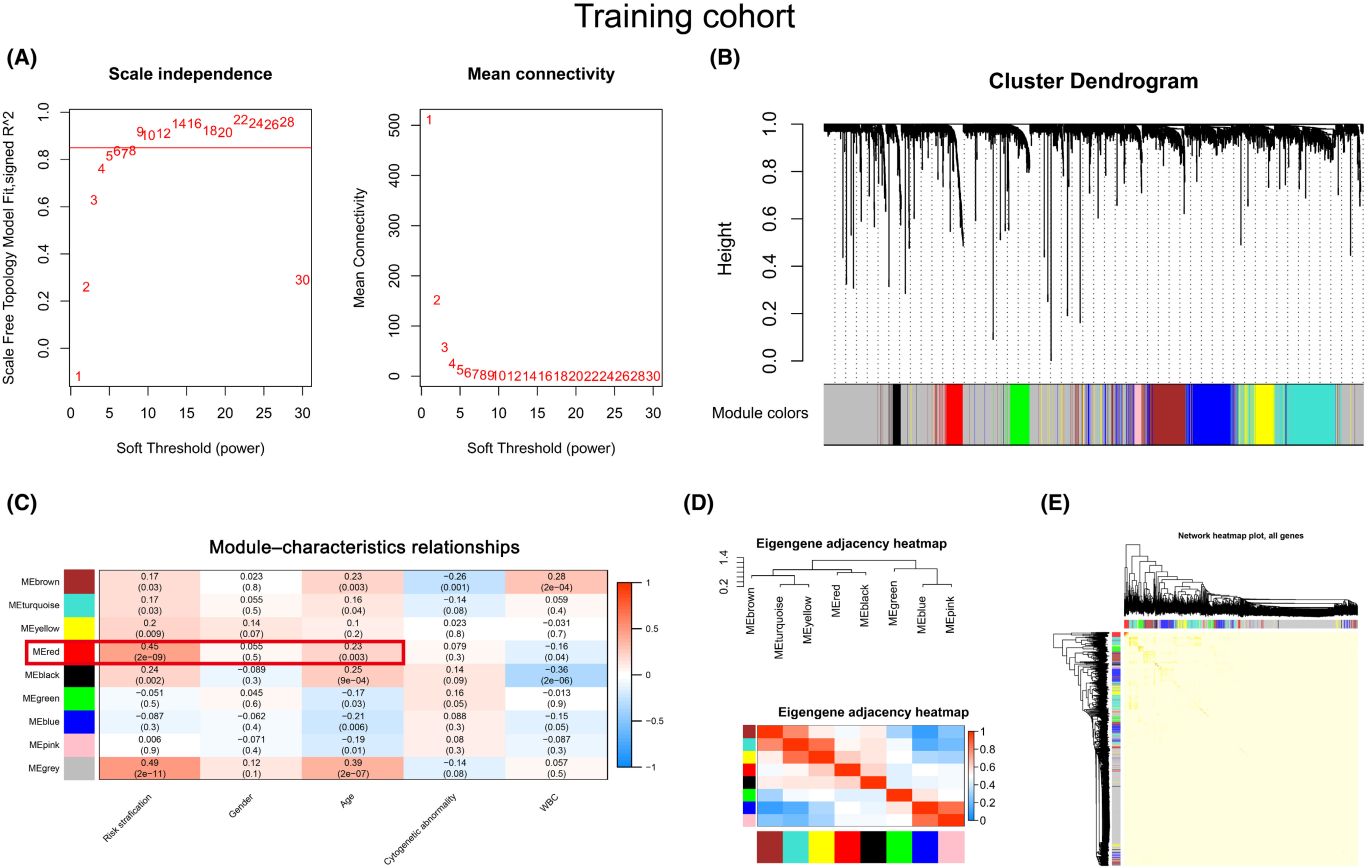
Identification of a module related to the clinical characteristics of AML by WGCNA. (A) A soft threshold (power) was obtained. The red line indicates that the scale‐free topology model was fitted to 0.85. (B) The gene dendrogram was constructed by hierarchical clustering. The different colors below the dendrogram display the modules corresponding to the co‐expressed genes. (C) Module–characteristics relationships. The rows represent the module eigengene (ME) and its color, and the column represents the clinical characteristics. The correlation coefficient and *p*‐value are shown in each cell. The red box shows the correlation between the red module, risk stratification, and age. (D) Heatmap of module adjacencies. Darker colors indicate a stronger correlation. (E) A topological overlapping heatmap of genes within the module. In the heatmap, darker colors indicate higher topological overlap.

To investigate the co‐expression patterns of lncRNAs and mRNAs in AML patients, 3,684 prognostic genes were included in constructing WGCNA in the training cohort. The soft threshold's power for constructing a scale‐free network was set to nine (Figure 1A). Then, a total of eight co‐expression modules, brown, turquoise, yellow, red, black, green, blue, and pink, were enriched, and their corresponding gene numbers were 277, 363, 58, 103, 49, 108, 300, and 43, respectively (Figure 1B). We further analyzed the relationships between modules and clinical characteristics and found that the red module was positively correlated with risk stratification (*r* = 0.45, *p* < 0.001) and age (*r* = 0.23, *p* = 0.003) (Figure 1C). A gene module is a cluster of densely interconnected genes in terms of co‐expression. WGCNA uses hierarchical clustering to identify gene modules and color to indicate modules. For genes that are not assigned to any of the modules, WGCNA places them in a gray module. That is, genes in the gray module are not co‐expressed.^14^, ^31^, ^32^, ^33^, ^34^ Although the gray module was positively correlated with risk stratification and age, and its correlations are stronger than the red module in Figure 1C, it may be due to the correlation between some independent genes in the gray module, rather than co‐expressed genes and risk differentiation and age; thus, the gray model was not chosen for further investigation and correlation with other modules in Figure 1D. In addition, we also investigated relationships between modules and found that the red module had a significant correlation with other modules (Figure 1D). Importantly, the genes within the red module were highly correlated (Figure 1E). Therefore, a total of 103 genes in the red module were selected for the following analysis.

### 3.2 High expression of LOC541471, STK25, SOD1, and GDAP1 was associated with poor OS in AML

**FIGURE 2:**
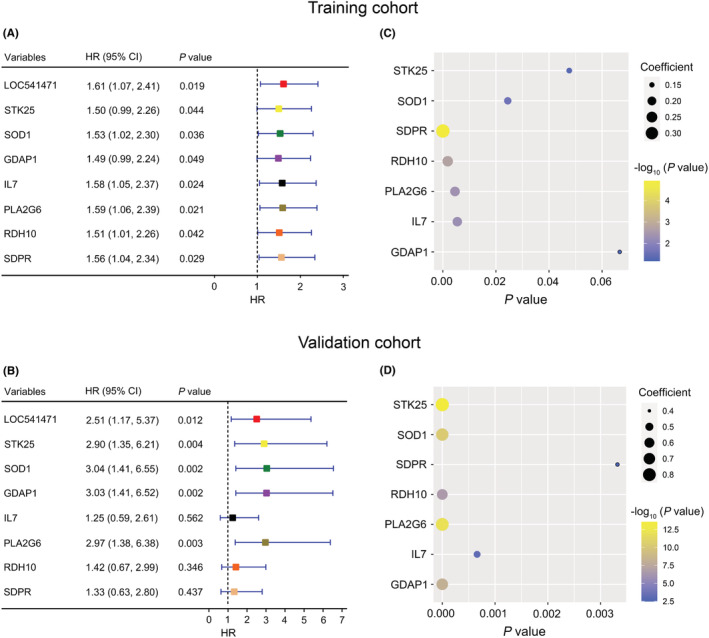
Kaplan–Meier survival analysis of co‐expressed genes. (A and B): Kaplan–Meier survival analysis of LOC541471, STK25, SOD1, GDAP1, IL7, PLA2G6, RDH10, and SDPR in the training (A) and validation (B) cohorts. (C and D): The bubble plots show the Spearman correlation coefficient and *P* values between LOC541471 and STK25, SOD1, GDAP1, IL7, PLA2G6, RDH10, and SDPR in the training (C) and validation (D) cohorts.

**FIGURE 3:**
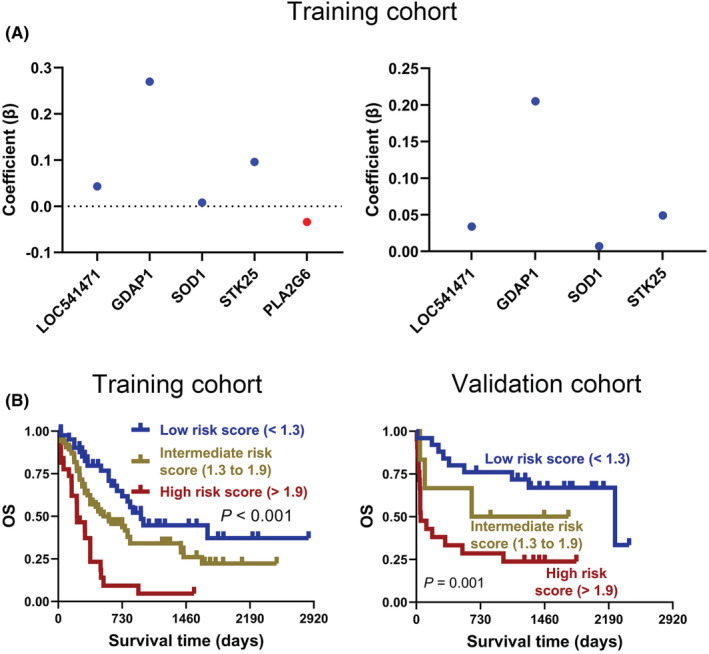
Weighted combination analysis of LOC541471 and co‐expressed mRNAs. (A) The multivariate COX regression coefficients for LOC541471 and co‐expressed mRNAs before (left) and after (right) adjustment in the training cohort. (B) The Kaplan–Meier for AML patients with low‐, intermediate‐, and high‐risk scores were plotted based on the risk scores 1.3 and 1.9 in training (left) and validation (right) cohorts.

After uni‐ and multivariate COX regression analysis, 64 genes were selected for subsequent Kaplan–Meier survival and protein–protein interaction (PPI) analysis in the training cohort (Figure S1A). As shown in Figure 2A and Figure S1B, there were 20 genes with a *p*‐value <0.05 in the Kaplan–Meier survival curves analysis. In addition, PPI analysis suggested that 27 genes have protein–protein interactions (Figure S1C). Thus, eight genes, LOC541471, STK25, SOD1, SDPR, RDH10, PLA2G6, IL7, and GDAP1, were included in the overlap of multivariate COX regression, Kaplan–Meier survival curve analysis, and PPI + LOC541471 (Figure S1D and Table S3). Importantly, high expression of LOC541471, STK25, SOD1, PLA2G6, and GDAP1 was related to poor OS (*p* < 0.05), whereas the expression levels of SDPR, RDH10, and IL7 were not significantly associated with OS by Kaplan–Meier survival analysis in the validation cohort (*p* > 0.05) (Figure 2B). Interestingly, the lncRNA LOC541471 was shown to be positively linked with STK25, SOD1, SDPR, RDH10, PLA2G6, IL7, and GDAP1 by Spearman correlation in the training cohort (*p* < 0.10, Figure 2C). This finding was supported by the validation cohort (*p* < 0.01, Figure 2D). Therefore, LOC541471, STK25, SOD1, PLA2G6, and GDAP1 were selected for multivariate COX regression analysis. However, PLA2G6 had the opposite contribution to OS in the uni‐ and multivariate COX regression models; thus, it was removed from the gene combination (Figure 3A, left panel). Finally, the coefficients in the multivariate COX regression model were used to calculate a risk score. Risk score = β_i_ * (gene expression level_i_) (Figure 3A, right panel). To further explore the impacts of the expression levels of LOC541471, STK25, SOD1, and GDAP1 on the RFS of AML patients, we performed Kaplan–Meier survival analysis in the validation cohort, and found that the expression levels of these four genes had no significant correlation with RFS (*p* > 0.05, Figure S2).

To investigate the prognostic value of risk score, two optimal prognostic cut‐off values of 1.3 and 1.9 were first calculated using X‐tile software, which divided the 167 AML patients in the training cohort into low‐, intermediate‐, and high‐risk score groups (Figure S3A). Notably, Kaplan–Meier analysis suggested that intermediate‐ and high‐risk patients were associated with poorer OS than low‐risk patients in the training cohort (*p* < 0.001, Figure 3B, left panel). Moreover, the validation cohort corroborated this finding (*p* = 0.001, Figure 3B, right panel).

### 3.3 Construction of a nomogram displaying and customizing the AML patients' overall survival rate

**FIGURE 4:**
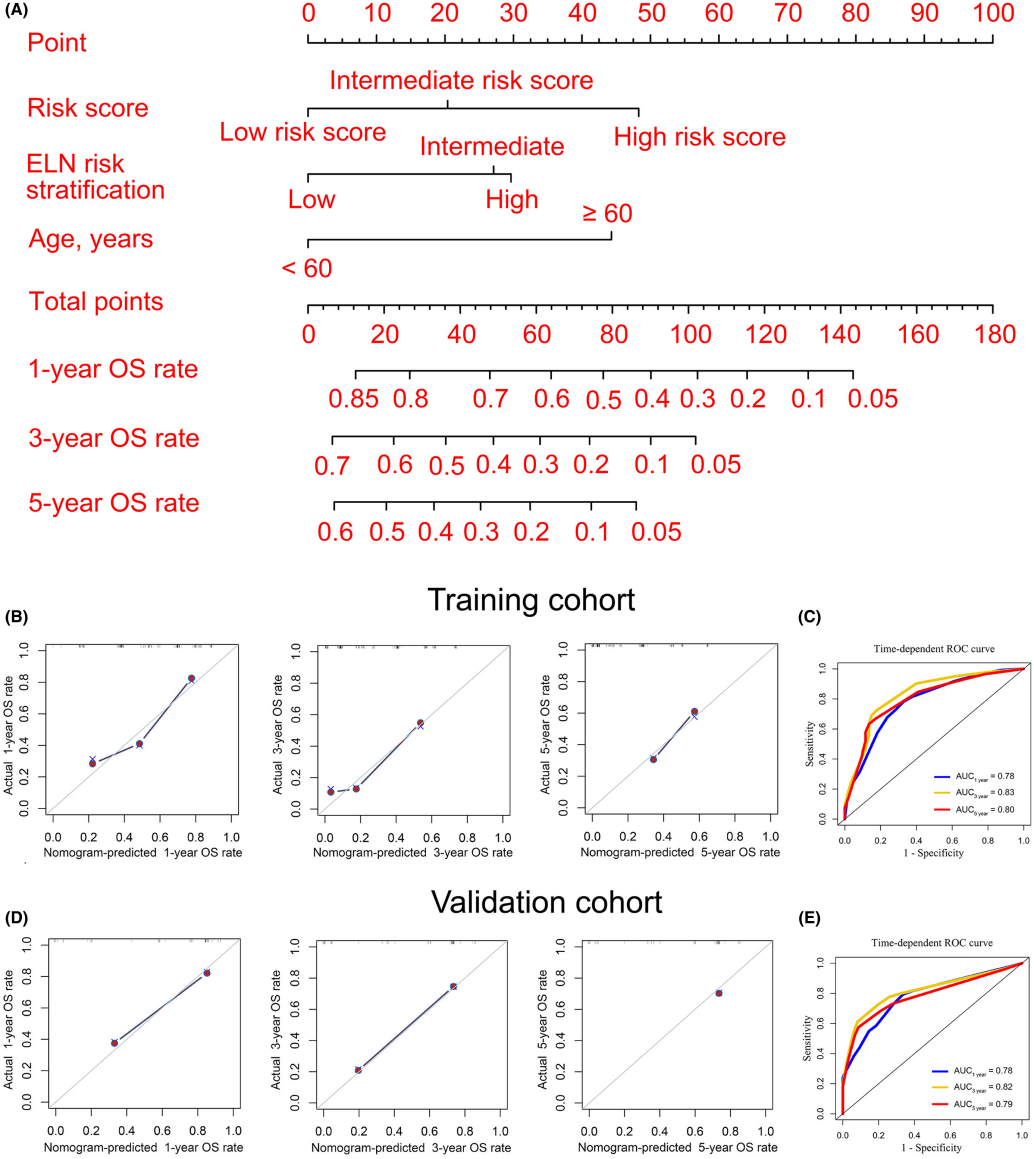
Nomogram to visualize and personalize the OS rate for AML patients. (A) Construction of a nomogram model. In general, each covariate of an individual contributes a point based on the evaluation of the nomogram model. Total points are obtained by adding the given points for all covariates. Then, the total points corresponding to the 1‐, 3‐, and 5‐year OS rates can be represented by the nomogram model. A higher total point usually indicates a lower expected OS rate. B and D: 1‐ (left), 3‐ (middle), and 5‐year (right) calibration curves for the nomogram model in training (B) and validation (D) cohorts. (C and E): Time‐dependent receiver operating characteristic (ROC) curves were used to evaluate the predictive performance of the nomogram model in the training (C) and validation (E) cohorts.

Univariate and multivariate COX regression analysis were used to select clinically significant variables for constructing a nomogram model. The results demonstrated that the risk score and ELN risk stratification were associated with poor OS and were independent prognostic factors for AML patients in the training cohort (hazard ratio (HR) >1, *p* < 0.05). Furthermore, for AML patients in the validation cohort, the risk score and age were independent prognostic factors (HR >1, *p* < 0.05) (Table 1). Moreover, AML patients over the age of 60 had a poorer prognosis than AML patients under the age of 60 (Figure S4). As a result, a nomogram model based on the risk score, ELN risk stratification, and age could be used to visualize and customize AML patients' 1‐, 3‐, and 5‐year OS rates (Figure 4A). Table S4 shows the points in the nomogram model for the variables and OS rates. The training and validation cohorts were also used to monitor the efficiency of the nomogram model in predicting OS. Calibration curves and time‐dependent ROC curves were used to evaluate the performance of the nomogram model in both training and validation cohorts. The result showed that the 1‐, 3‐, and 5‐year OS rates predicted by the nomogram were highly consistent with actual observations in the training cohort (Figure 4B). Moreover, according to the time‐dependent ROC curve in the training cohort, the AUCs were 0.78 (Figure 4C). More importantly, these findings were verified again in the validation cohort (Figure 4D, E). Taken together, the above data indicate that the nomogram model we constructed performs well in predicting the survival rate of AML patients.

**TABLE 1:** Uni‐ and multivariate regression analysis in AML patients.

Variables	Univariate COX regression				Multivariate COX regression			
	Training cohort		Validation cohort		Training cohort		Validation cohort	
	HR (95% CI)	*p* Value	HR (95% CI)	*p* Value	HR (95% CI)	*p* Value	HR (95% CI)	*p* Value
Risk score								
Low (<1.3)	reference		reference		reference		reference	
Intermediate (1.3 to 1.9)	1.91 (1.15, 3.18)	**0.013**	1.93 (0.51, 7.28)	0.334	1.66 (1.00, 2.77)	0.052	1.13 (0.28, 4.60)	0.869
High (>1.9)	4.70 (2.59, 8.51)	**< 0.001**	4.31 (1.82, 10.17)	**0.001**	3.05 (1.65, 5.64)	**< 0.001**	2.70 (1.05, 6.97)	**0.039**
ELN risk stratification								
Low	reference		reference		reference			
Intermediate	2.48 (1.34, 4.61)	0.004	2.07 (0.47, 9.22)	0.338	1.88 (1.00, 3.53)	0.051		
High	3.63 (1.81, 7.26)	<0.001	3.21 (0.59, 17.57)	0.179	2.09 (1.02, 4.31)	0.045		
Age, years	1.04 (1.03, 1.06)	<0.001	1.06 (1.03, 1.09)	< 0.001	1.04 (1.02, 1.05)	<0.001	1.04 (1.01, 1.07)	0.018
Gender								
Female	reference		reference					
Male	0.98 (0.67, 1.44)	0.934	1.50 (0.71, 3.17)	0.283				
Cytogenetic abnormality								
No	reference		reference					
Yes	0.92 (0.61, 1.38)	0.682	1.00 (0.39, 2.55)	0.998				
White blood cells	1.00 (1.00, 1.01)	0.054	1.01 (1.00, 1.01)	< 0.001			1.00 (1.00, 1.01)	0.243

Abbreviations: CI, confidence interval; ELN, European Leukemia Network; HR, hazard ratio.

### 3.4 Establishment of risk stratification for AML patients

**FIGURE 5:**
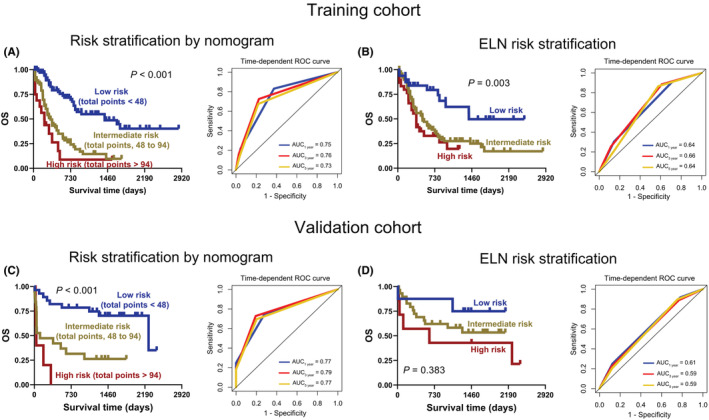
Establishment of risk stratification for AML patients. A and C: The Kaplan–Meier (left) and time‐dependent ROC curves (right) for AML patients with low, intermediate, and high risk were plotted according to the total points 48 and 94 in the training (A) and validation (C) cohorts. B and D: Kaplan–Meier (left) and time‐dependent ROC curves (right) of AML patients were constructed based on existing European Leukemia Network (ELN) risk stratification in the training (B) and validation (D) cohorts.

To perform risk stratification, two optimal cut‐off values for total points were first determined using X‐tile software, 48 and 94, respectively, which divided the 167 AML patients in the training cohort into three groups: low ‐, intermediate‐, and high‐risk groups (Figure S3B). Importantly, the Kaplan–Meier and ROC curves suggested that the risk stratification constructed by nomogram was as effective as the existing European Leukemia Network (ELN) risk stratification in the training cohort (risk stratification by nomogram vs. ELN, *p* < 0.001 vs. *p* = 0.003, AUC ≥0.73 vs. ≥ 0.64) (Figure 5A, B). Notably, the risk stratification developed by nomogram had better performance in predicting OS than the existing ELN risk stratification in the validation cohort (risk stratification by nomogram vs. ELN, *p* < 0.001 vs. *p* = 0.383, AUC ≥0.77 vs. ≥ 0.59) (Figure 5C, D).

### 3.5 Knockdown of LOC541471, GDAP1, SOD1, or STK25 promotes apoptosis and inhibits proliferation

**FIGURE 6:**
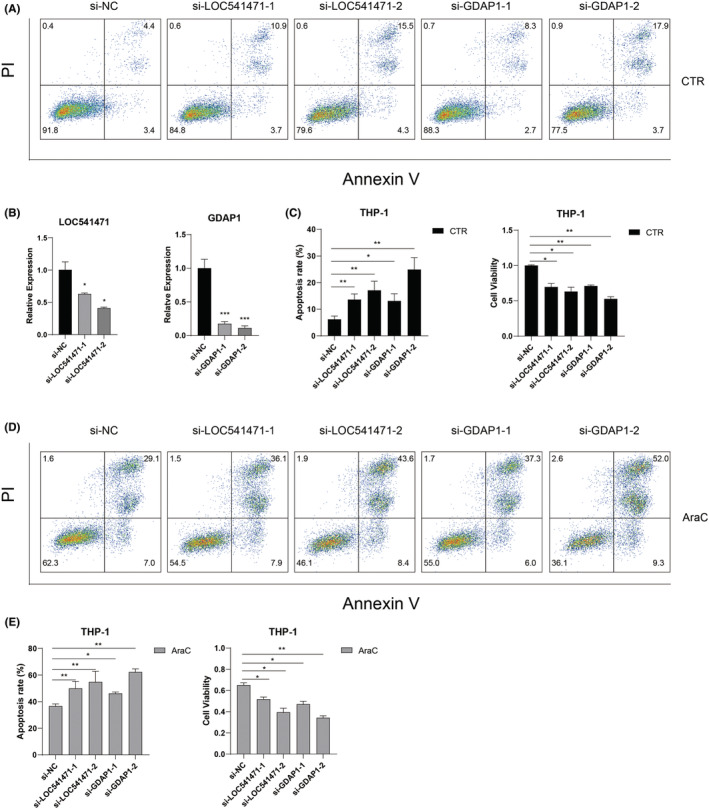
Knockdown of LOC541471 or GDAP1 promotes apoptosis and inhibits proliferation in AML cells. (A) Representative plots of the apoptosis of THP‐1 cells detected by flow cytometry. THP‐1 cells were transfected with control siRNA, LOC541471 siRNA, or GDAP1 siRNA. (B) Quantitative real‐time RT‐PCR (qRT‐PCR) was used to detect the expression level of LOC541471 or GDAP1 in THP‐1 cells after knocking down LOC541471 or GDAP1. 18 S rRNA served as an internal control. (C) The apoptosis rate and cell viability of THP‐1 cells transfected with corresponding siRNAs in three repeated experiments were analyzed. (D) Representative plots of the apoptosis of THP‐1 cells transfected with corresponding siRNAs followed by a 24 h AraC (1 μM) treatment. (E) The apoptosis rate and cell viability of THP‐1 cells transfected with corresponding siRNAs followed by a 24 h AraC (1 μM) treatment.

Because the high expression of LOC541471, GDAP1, SOD1, and STK25 has poor OS in AML, we further investigated the function of these four genes in AML cells. Specific siRNAs knocked down LOC541471 and GDAP1 to observe their impact on apoptosis and proliferation. After transduction of si‐LOC541471 or si‐GDAP1 into THP‐1 cells followed by treatment with AraC for 24 hours, loss of LOC541471 or GDAP1 significantly increased the rate of apoptosis and inhibited the viability of cells with or without AraC treatment when compared to control cells (Figure 6). In addition, the silence of SOD1 or STK25 could promote AraC‐induced apoptosis and inhibit cell proliferation (Figure S5). Overall, these results suggested that LOC541471, GDPA1, SOD1, or STK25 might serve as oncogenes in AML and their alteration may influence the clinical outcome of AML patients.

## 4 DISCUSSION

It is known that risk stratification has great significance in monitoring AML patients and guiding clinical treatment.^6^, ^35^, ^36^ However, AML patients still have a bad prognosis.^9^ Thus, it is critical to explore new biomarkers for more precise risk stratification and predicting prognosis for patients. In this study, based on the transcriptome, we found that high expression of LOC541471, GDAP1, SOD1, and STK25 was found to be strongly related to a poor prognosis in AML patients. Furthermore, the weighted combination of these four genes could lead to risk stratification of patients, which acted as an independent prognostic predictor for AML patients. This finding might provide a reference for improving the existing ELN risk stratification.

Various studies have found that high expression of lncRNA LOC541471, also known as MIR4435‐2HG, promotes cell growth and metastasis in various malignancies, including glioblastoma and ovarian cancer.^37^, ^38^, ^39^ Additionally, LOC541471 could be a possible biomarker for cancer patients' prognosis.^40^, ^41^ This finding was consistent with our results, that is, high LOC541471 was correlated with poor prognosis in AML patients. In addition, knockdown of LOC541471 could promote AML apoptosis and inhibit cell proliferation. GDAP1 encodes a ganglioside‐induced differentiation‐related protein that may play a critical role in signal transduction during neuron development.^42^ However, the importance of GDAP1 in cancers, including AML, has not yet been revealed. This study suggests that AML patients with up‐regulated GDAP1 have poor OS, and silencing GDAP1 could promote apoptosis and inhibit the proliferation of AML cells. As expected, high expression of SOD1 is significantly correlated with poor prognosis for a variety of cancers, and downregulation of SOD1 can inhibit the growth of cancer cells.^43^, ^44^ Moreover, it was reported that SOD1 inhibition decreases cell growth and induces cell death in myeloid leukemia cells.^45^ These findings are similar to our results, that is, silence of SOD1 could promote AraC‐induced apoptosis and inhibit AML cell proliferation, and high SOD1 was related to poor prognosis in AML patients. Notably, the Gene Expression Profiling Interactive Analysis (GEPIA) database (available at: http://gepia.cancer‐pku.cn/) shows that high STK25 expression has unfavorable prognoses in adrenocortical carcinoma, liver hepatocellular carcinoma, mesothelioma, and uveal melanoma (unpublished data). These results were consistent with our results that high STK25 corrected with poor prognosis for AML patients and silence of STK25 could promote AraC‐induced apoptosis and inhibit AML cell proliferation. These findings indicate that LOC541471, GDAP1, SOD1, and STK25 might have potentially developed risk stratification for AML patients.

Nomogram models can display and personalize the prognosis of cancer patients, providing a new perspective for evaluating prognosis and patient monitoring.^20^, ^21^ In this study, a nomogram model based on LOC541471, GDAP1, SOD1, and STK25 risk scores, ELN risk stratification, and age could visualize and customize the 1‐, 3‐, and 5‐year OS rates of AML patients. The nomogram model was further evaluated using calibration and time‐dependent ROC curves from the training and validation cohorts, indicating that the nomogram model performed better in predicting the OS rate of AML patients. Importantly, a new risk stratification was developed for AML patients based on the nomogram model, which could divide AML patients into low‐, intermediate‐ and high‐risk groups.

However, the lack of a large number of AML samples from multiple centers to validate the universal applicability of the risk stratification that we developed, as well as the nomogram model's accuracy in predicting the OS rate, are two of the study's limitations. Moreover, the detailed role and further in vivo results of LOC541471, GDAP1, SOD1, and STK25 in AML need further exploration.

## 5 CONCLUSION

We demonstrate that high expression of LOC541471, GDAP1, SOD1, and STK25 was related to poor OS of AML patients. Next, the 1‐, 3‐, and 5‐year OS rates of AML patients were demonstrated and personalized using a nomogram model that included risk score, ELN risk stratification, and age. Furthermore, a new risk stratification system based on the nomogram model was established for AML patients. Importantly, preliminary findings indicated that silencing LOC541471, GDAP1, SOD1, or STK25 promotes apoptosis and inhibits the proliferation of AML cells. This discovery will provide novel insight into evaluating prognosis, patient monitoring, and designing combinational targeted therapy for AML.

## AUTHOR CONTRIBUTIONS

**Xibao Yu:** Data curation (equal); formal analysis (equal); funding acquisition (equal); methodology (equal); writing – original draft (equal); writing – review and editing (equal). **Cunte Chen:** Data curation (equal); formal analysis (equal); methodology (equal); writing – original draft (equal); writing – review and editing (equal). **Yanyun Hu:** Data curation (equal); formal analysis (equal); methodology (equal); writing – original draft (equal); writing – review and editing (equal). **Kehan Li:** Formal analysis (supporting). **Yikai Zhang:** Formal analysis (supporting). **Zheng Chen:** Formal analysis (supporting). **Dingrui Nie:** Formal analysis (supporting). **Rili Gao:** Formal analysis (supporting). **Youxue Huang:** Formal analysis (supporting). **Mengjun Zhong:** Formal analysis (supporting). **Caixia Wang:** Funding acquisition (supporting); methodology (equal); resources (equal). **Shun‐Qing Wang:** Methodology (equal); resources (equal). **Yi‐Xin Zeng:** Conceptualization (equal); project administration (equal); supervision (equal); writing – original draft (equal); writing – review and editing (equal). **Yangqiu Li:** Conceptualization (equal); project administration (equal); supervision (equal); writing – original draft (equal); writing – review and editing (equal). **chengwu zeng:** Conceptualization (equal); funding acquisition (equal); project administration (equal); supervision (equal); writing – original draft (equal); writing – review and editing (equal).

## FUNDING INFORMATION

This study was supported by grants from the National Natural Science Foundation of China (Nos. 81770158, 81500126, and 82200167), the Guangzhou Science and Technology Project (Nos. 201807010004, 201803040017, and 201904010033), the Pearl River S&T Nova Program of Guangzhou, China (No. 201906010002), the Guangdong Basic and Applied Basic Research Foundation (No. 2021A1515110140), and the Guangdong Natural Science Foundation (No. 2022A1515012478).

## CONFLICT OF INTEREST

The authors declare that the research was conducted in the absence of any commercial or financial relationships that could be construed as a potential conflict of interest.

## ETHICS APPROVAL

This study was approved by the Ethics Committee of the First People's Hospital of Guangzhou, and all participants provided written informed consent.

## Supporting information


Data S1

## Data Availability

The datasets used and analyzed during the current study are available upon reasonable request from the corresponding author.
