# Supplementary material for: High expression of LOC541471, GDAP1, SOD1, and STK25 is associated with poor overall survival of patients with acute myeloid leukemia
Source: Cancer Med. 2023 Jan 27;12(7):9055–67. doi: 10.1002/cam4.5644 (PMC10134312; doi:10.1002/cam4.5644)
Supplement: Supplementary file 1 — Data S1 [file CAM4-12-9055-s001.pdf]

Table S1. Clinical characteristics of AML patients.

Variables	Training cohort, n (%) [a]	Validation cohort, n (%) [a]	*P* value
Number	167	52	NA
Age, mean ± SD, years	56 ± 16	42 ± 16	< 0.001
Gender, n (%)			0.618
Male	89 (53.3)	25 (48.1)	
Female	78 (46.7)	27 (51.9)	
Cytogenetic abnormality			0.153
Yes	69 (41.3)	17 (32.7)	
No	84 (50.3)	26 (50.0)	
Unknown	14 (8.4)	9 (17.3)	
WBC, mean ± SD, ($\times 10^9$/L)	34.5 ± 43.2	44.7 ± 62.4	0.623
Subtype			< 0.001
M0	15 (9.0)	0 (0.0)	
M1	36 (21.6)	1 (1.9)	
M2	37 (22.2)	17 (32.7)	
M3	16 (9.6)	3 (5.8)	
M4	35 (21.0)	5 (9.6)	
M5	21 (12.6)	20 (38.5)	
M6	2 (1.2)	2 (3.8)	
M7	3 (1.8)	0 (0.0)	
Unclassified	2 (1.2)	4 (7.7)	
ELN risk stratification			< 0.001
Low	33 (19.8)	8 (15.4)	
Intermediate	97 (58.1)	29 (55.8)	
High	35 (20.9)	7 (13.5)	
Unknown	2 (1.2)	8 (15.4)	
Treatment			NA
Chemotherapy	NA	29 (55.8)	
Allo-HSCT	NA	23 (44.2)	

[a] Unless specifically mentioned; ELN, European Leukemia Network; HSCT, Allogeneic hematopoietic stem cell transplantation; NA, not available; SD, standard deviation; WBC, white blood cell.

Table S2. Primers for qRT-PCR and siRNA sequence.

**Targets**	**Sequence 5' - 3'**
LOC541471 (F)	ATGTCGGGAGAGGAAGTGGT
LOC541471 (R)	CTTCCCAGGAACTGTGCTGT
GDAP1 (F)	ATGCGTTTGAACTCAACTGGA
GDAP1 (R)	TCAGGCATTAACCTGGGTGTT
IL7 (F)	GGACTTCCTCCCCTGATCCT
IL7 (R)	TCGATGCTGACCATTAGAACACT
PLA2G6 (F)	TTTGGCCGCCTGGTCAATAC
PLA2G6 (R)	CTCCCGAACTCGGTCACTC
RDH10 (F)	GAAAAGCCTTAGTGGTCCAGAAG
RDH10 (R)	ACCTGACGGCTGAAAGAGTC
SDPR (F)	AAGAGCGCATGGATAGGCAG
SDPR (R)	TCATCGTGGGGCAAATCATCA
SOD1 (F)	GGTGGGCCAAAGGATGAAGAG
SOD1 (R)	CCACAAGCCAAACGACTTCC
STK25 (F)	CTCCGGGGATTTGCCAACC
STK25 (R)	TAGGTAGGAGCCAAAGTAGCG
18S rRNA (F)	CGGCGGCTTTGGTGACTCTAGA
18S rRNA (R)	CCTGCTGCCTTCCTTGGATGTG
si-LOC541471-1	GCACAGAGCUUUCCCUUUAUC
si-LOC541471-2	GCAUGGAACUCGACAGUUA
si-GDAP1-1	GGCCACUCAGAUCAUUGAUUAUCUU
si-GDAP1-2	CACUCGCUGUCACAUUGCAUCGACU
si-SOD1	GACUUGGGCAAAGGUGGAA
si-STK25	GCAUCGAUAACCACACAAA

Table S3. Abbreviation for genes.

Genes	Type	Full or other name
LOC541471	lncRNA	MIR4435-2HG
GDAP1	mRNA	Ganglioside induced differentiation associated protein 1
IL7	mRNA	Interleukin 7
PLA2G6	mRNA	Phospholipase A2 group VI
RDH10	mRNA	Retinol dehydrogenase 10
SDPR	mRNA	Serum deprivation protein response
SOD1	mRNA	Superoxide dismutase 1
STK25	mRNA	Serine/threonine kinase 25

Table S4. The points for nomogram model.

Variable	Points	Survival time	OS rate	Total points
Risk score		1 year		
Low (< 1.3)	0		0.85	13
Intermediate (1.3 to 1.9)	20		0.80	27
High (> 1.9)	48		0.70	48
ELN risk stratification			0.60	64
Low	0		0.50	78
Intermediate	27		0.40	90
High	30		0.30	102
Age			0.20	115
< 60 years	0		0.10	131
≥ 60 years	44		0.05	143
		3 year		
			0.70	6
			0.60	23
			0.50	36
			0.40	49
			0.30	61
			0.20	74
			0.10	90
			0.05	102
		5 year		
			0.60	7
			0.50	21
			0.40	33
			0.30	45
			0.20	58
			0.10	74
			0.05	86

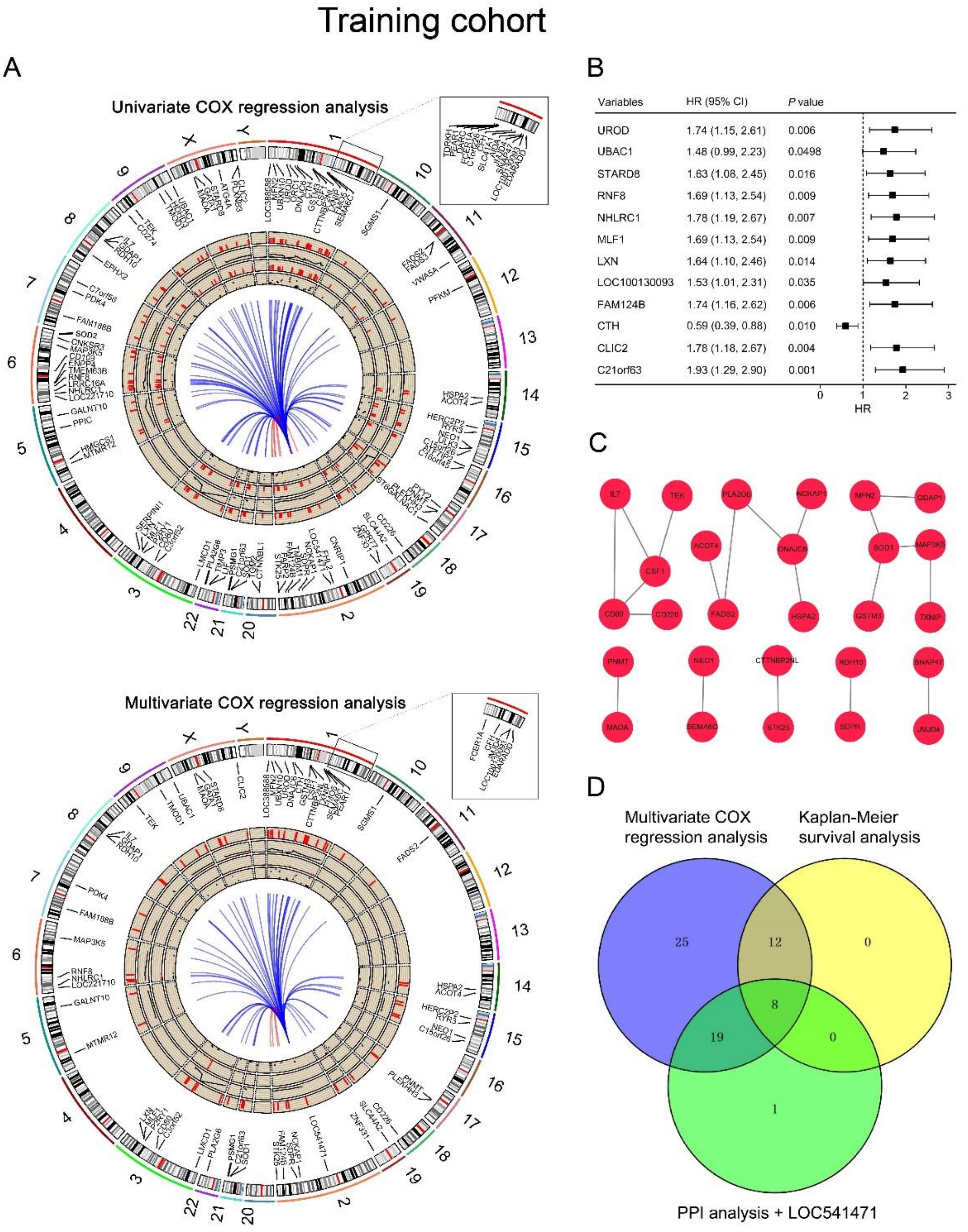

Fig. S1. Identification of prognosis genes. (A) Analysis of prognostic genes in the red module by uni- and multivariate COX regression models. (upper panel) Univariate COX regression analysis of 103 genes in the red module. (bottom panel) 64 genes after multivariate COX regression analysis are shown. The outermost layer is a human chromosome map with black and white bars indicating chromosomal bands and red bars indicating centromeres. The

second layer represents the lower value of the 95% confidence interval for the hazard ratio. The line plot shows the hazard ratio in the third layer. The fourth layer represents the upper value of the 95% confidence interval for the hazard ratio. The innermost layer exhibits the *P*-value, which was <0.05. The network at the center indicates mRNAs co-expressed with LOC541471; red lines indicate mRNAs on the same chromosome as LOC541471, and blue lines indicate mRNAs on different chromosomes. (B) Kaplan-Meier survival analysis of independent prognostic genes in the red module after multivariate COX regression analysis. Forest plots of 12 genes with a *P*-value < 0.05 are shown. (C) Protein-protein interaction (PPI) analysis of the 64 independent prognostic genes using the STRING database (https://string-db.org/). (D) The intersection of multivariate COX regression analysis, Kaplan-Meier survival analysis, and PPI+LOC541471.

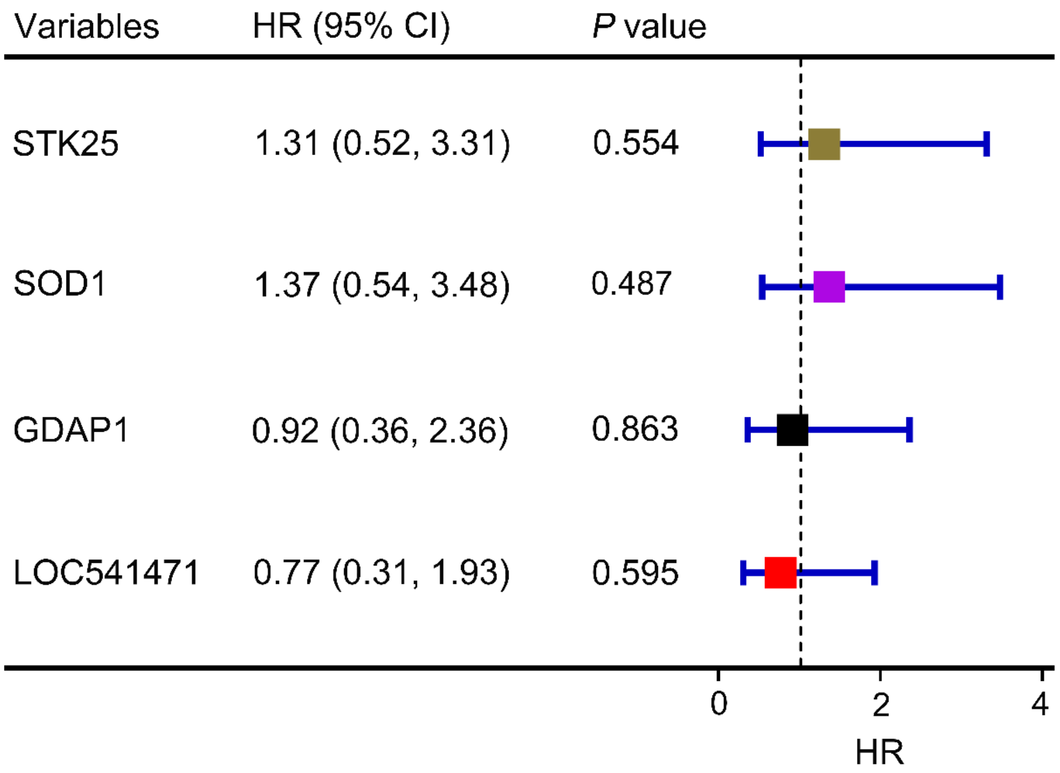

Fig. S2. Kaplan-Meier survival analysis of LOC541471, STK25, SOD1, and GDAP1 in the validation cohort.

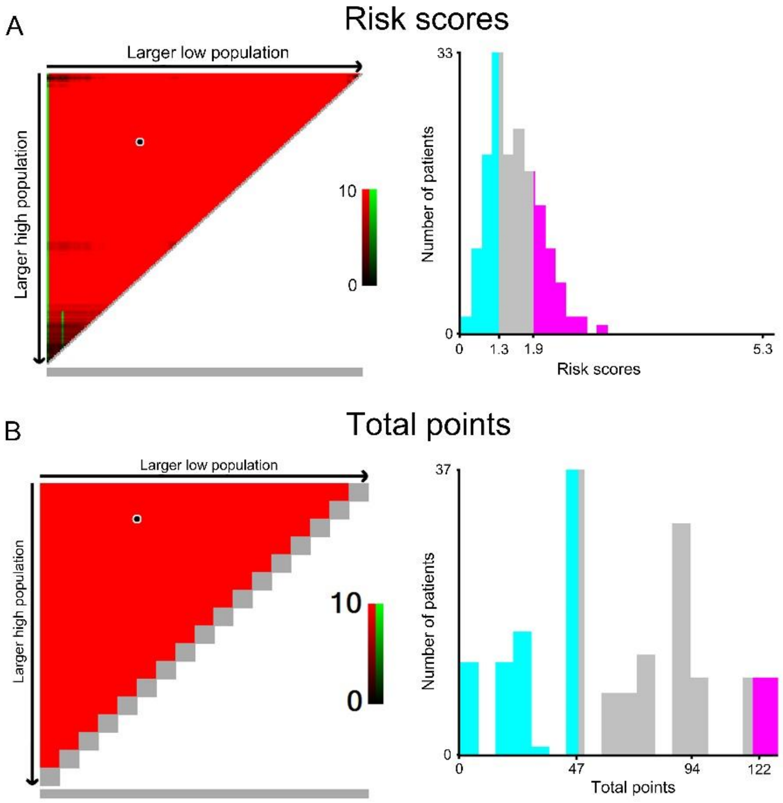

Fig. S3. X-tile software (version 3.6.1, Yale University, New Haven, CT, USA) was used to obtain the optimal prognostic cut-off value for risk scores (A) and total points (B) in the training cohort. The black dots represent the distribution of the AML population at two optimal cut-off values of risk scores or total points (left panel). A higher red color or a lower green color represents a higher risk score or total points. Distribution of AML patients at different risk scores or total points (right panel).

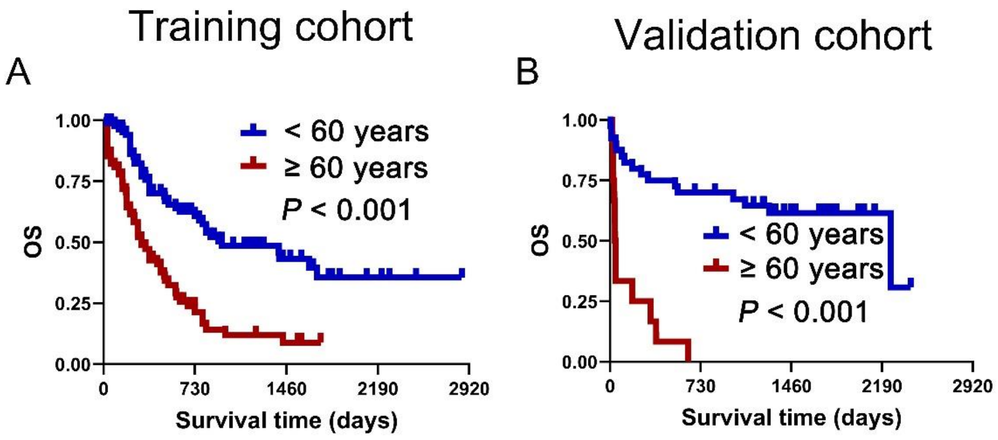

Fig. S4. Kaplan-Meier curves of AML patients according to age < 60 and ≥ 60 in the training (A) and validation (B) cohorts.

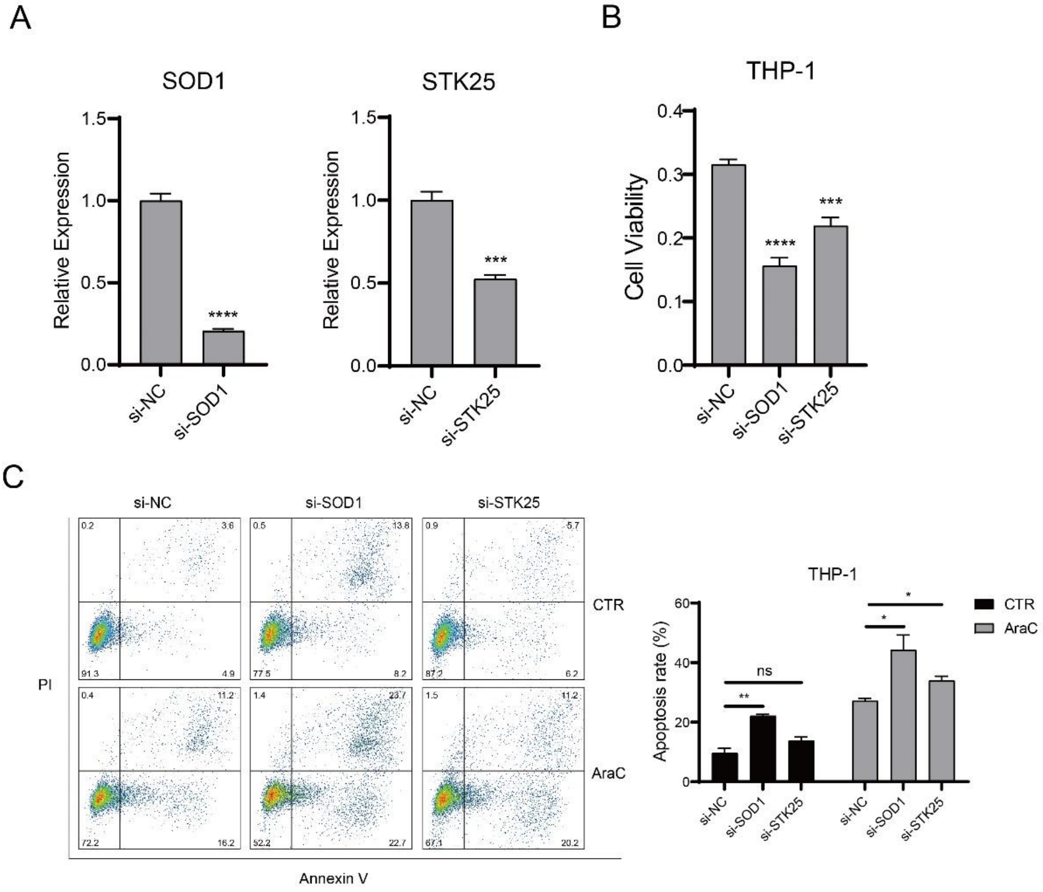

Fig. S5. Silence of SOD1 or STK25 promotes AraC-induced apoptosis and inhibits cell proliferation. (A) qRT-PCR was used to detect the expression level

of SOD1 or STK25 in THP-1 cells after knocking down SOD1 or STK25. 18S rRNA served as an internal control. (B) Cell viability of THP-1 cells in three repeated experiments were analyzed. THP-1 cells were transfected with control siRNA, SOD1 siRNA, or STK25 siRNA followed by a 48 h AraC (1 μM) treatment. (C) Representative plots of the apoptosis of THP-1 cells detected by flow cytometry and the apoptosis rate in three repeated experiments were analyzed. THP-1 cells were transfected with control siRNA, SOD1 siRNA, or STK25 siRNA followed by a 24 h AraC (1 μM) treatment.
